# Inhibition of HDAC3- and HDAC6-Promoted Survivin Expression Plays an Important Role in SAHA-Induced Autophagy and Viability Reduction in Breast Cancer Cells

**DOI:** 10.3389/fphar.2016.00081

**Published:** 2016-03-31

**Authors:** Jane Ying-Chieh Lee, Ching-Wen Kuo, Shing-Ling Tsai, Siao Muk Cheng, Shang-Hung Chen, Hsiu-Han Chan, Chun-Hui Lin, Kun-Yuan Lin, Chien-Feng Li, Jagat R. Kanwar, Euphemia Y. Leung, Carlos Chun Ho Cheung, Wei-Jan Huang, Yi-Ching Wang, Chun Hei Antonio Cheung

**Affiliations:** ^1^Department of Pharmacology, College of Medicine, National Cheng Kung UniversityTainan, Taiwan; ^2^Institute of Basic Medical Sciences, College of Medicine, National Cheng Kung UniversityTainan, Taiwan; ^3^Division of Hematology and Oncology, Department of Internal Medicine, Chi-Mei Medical CenterLiouying, Tainan, Taiwan; ^4^Department of Pathology, Chi-Mei Medical Center, Yung Kang DistrictTainan, Taiwan; ^5^Nanomedicine-Laboratory of Immunology and Molecular Biology, Faculty of Health, School of Medicine, Deakin UniversityWaurn Ponds, Geelong, VIC, Australia; ^6^Auckland Cancer Society Research Centre and Department of Molecular Medicine and Pathology, University of AucklandAuckland, New Zealand; ^7^School of Biological Sciences, Faculty of Science, University of AucklandAuckland, New Zealand; ^8^Graduate Institute of Pharmacognosy, Taipei Medical UniversityTaipei, Taiwan

**Keywords:** HDAC, XIAP, SAHA, survivin, breast cancer

## Abstract

SAHA is a class I HDAC/HDAC6 co-inhibitor and an autophagy inducer currently undergoing clinical investigations in breast cancer patients. However, the molecular mechanism of action of SAHA in breast cancer cells remains unclear. In this study, we found that SAHA is equally effective in targeting cells of different breast cancer subtypes and tamoxifen sensitivity. Importantly, we found that down-regulation of survivin plays an important role in SAHA-induced autophagy and cell viability reduction in human breast cancer cells. SAHA decreased survivin and XIAP gene transcription, induced survivin protein acetylation and early nuclear translocation in MCF7 and MDA-MB-231 breast cancer cells. It also reduced survivin and XIAP protein stability in part through modulating the expression and activation of the 26S proteasome and heat-shock protein 90. Interestingly, targeting HDAC3 and HDAC6, but not other HDAC isoforms, by siRNA/pharmacological inhibitors mimicked the effects of SAHA in modulating the acetylation, expression, and nuclear translocation of survivin and induced autophagy in MCF7 and MDA-MB-231 cancer cells. Targeting HDAC3 also mimicked the effect of SAHA in up-regulating the expression and activity of proteasome, which might lead to the reduced protein stability of survivin in breast cancer cells. In conclusion, this study provides new insights into SAHA's molecular mechanism of actions in breast cancer cells. Our findings emphasize the complexity of the regulatory roles in different HDAC isoforms and potentially assist in predicting the mechanism of novel HDAC inhibitors in targeted or combinational therapies in the future.

## Introduction

HDAC inhibitors, such as suberoylanilide hydroxamic acid (SAHA, Vorinostat) and Trichostatin A (TSA), have been shown to exhibit pleiotropic anticancer activities in many preclinical and clinical investigations of human cancers (Vigushin et al., 2001; Kelly et al., 2003; Roh et al., 2004; Condorelli et al., 2008). Among different HDAC inhibitors, SAHA is a class I HDAC/HDAC6 co-inhibitor that has already been approved by the U.S. Food and Drug Administration (FDA) for the treatment of advanced cutaneous T-cell-lymphoma. Its safety and effectiveness are also currently undergoing various Phase I/II clinical evaluations (*ClinicalTrials.gov* identifier: NCT00416130, NCT00368875, NCT01720602) in male/female patients with breast cancer. Surprisingly, although several studies have shown that SAHA induces autophagy, apoptosis, and exhibits potent anti-proliferative activity in cancer cells, the exact mechanisms by which SAHA induces these effects have not been fully understood (Butler et al., 2002; Lee et al., 2012).

Survivin is a well-known member of the inhibitor-of-apoptosis proteins (IAPs) family. It regulates mitosis and inhibits both caspase-dependent and -independent apoptosis in cancer cells (Li et al., 1998; Tamm et al., 1998; Cheung et al., 2010; Coumar et al., 2013). Interestingly, our previous study revealed that even though survivin is an inhibitor of apoptosis, targeting survivin by small molecule inhibitor or by siRNA induces autophagy and autophagic cell death in breast cancer cells regardless of the endogenous expression of p53 and caspase-3 (Cheng et al., 2015). However, survivin is traditionally classified as an apoptosis inhibitor; therefore, the role of survivin in SAHA-induced autophagy and autophagic cell death in cancer cells has seldom been investigated.

In this study, we found that SAHA down-regulates survivin expression at both transcriptional and post-transcriptional levels in part through HDAC2, HDAC3, and HDAC6 inhibitions. In addition, we found that down-regulation of survivin plays an important role in regulating SAHA induced autophagy and cell viability reduction in breast cancer cells.

## Materials and methods

### Cell lines and cell culture conditions

Human breast adenocarcinoma cell lines MCF7 (p53 wild-type), MDA-MB-231 (p53 mutant), and SK-BR-3 (p53 mutant) were originally obtained from ATCC (Table 1). Briefly, MCF7 cells were cultured in α-MEM containing 5% fetal bovine serum (FBS), penicillin/streptomycin/glutamine (PSG), and insulin transferrin selenium [ITS (Roche, cat# 11074547001)]. MDA-MB-231 cells were cultured in RPMI containing 10% FBS and PSG. SK-BR-3 cells were cultured in DMEM containing 10% FBS and PSG. All cell lines were incubated at 37°C in a humidified incubator containing 5% CO_2_ in air and were shown to be mycoplasma free. A series of MCF7-derived ER^+^/tamoxifen-resistant breast cancer cell lines (TamC3 and TamR8) were also used in this study. The cellular and molecular phenotypes of these tamoxifen-resistant breast cancer cell lines have already been characterized in a previous study (Leung et al., 2010). TamR8 breast cancer cells were cultured in α-MEM containing 5% fetal bovine serum (FBS), penicillin/streptomycin (10,000 unit/mL and 10 mg/mL, respectively), insulin transferring selenium (ITS, Roche), and tamoxifen (5 μM). In contrast, TamC3 breast cancer cells were cultured in phenol-red-free RPMI containing 5% charcoal-stripped FBS, penicillin/streptomycin (10,000 unit/mL and 10 mg/mL, respectively), and ITS (10 mg/L).

**Table 1:** **Characteristics of different cancer cell lines used in the study**.

**Cells**	**ER status**	**P53 status**	**Caspase-3 status**	**Tamoxifen sensitivity**	**SAHA IC_50_ (μM)**
MCF7	Expressing	Wild-type	Deficient	Sensitive	0.7 ± 0.1
MCF7-TamC3	Expressing	Wild-type	Deficient	Resistant	0.9 ± 0.1
MCF7-TamR8	Expressing	Wild-type	Deficient	Resistant	1.2 ± 0.3
MDA-MB-231	Deficient	Mutant	Expressing	Resistant	1.6 ± 0.5
Sk-Br-3	Deficient	Mutant	Expressing	Resistant	1.3 ± 0.5

*Breast cancer cells were treated with SAHA for 4 days and cell viability was determined by MTT assay. The IC_50_-values (inhibitory concentrations at which 50% decrease in cell viability is achieved) represent the average of at least three independent experiments*.

### MTT cell viability assay

A total of 5040 cells were seeded onto each well of 96-well plates for 24 h prior treating with SAHA for 96 h. After treatment, 180 μL of MTT solution [mixing MTT (Amresco, cat# 0793) 5 mg/mL in phenol-red free RPMI in a ratio of 1:10] was added to each well and incubated for 4 h. Then, 100 μL MTT lysis buffer was added to each well and incubated for 12 h. The absorbance of the solution was quantified by measuring at 570 nm wavelength by a spectrophotometer. The percentage cell growth inhibition for each treatment group was calculated by adjusting the untreated control group to 100%. All experiments were done using duplicate wells, and repeated at least three times.

### Gene silencing by siRNA

Target-validated siRNA oligos were transfected into MCF-7 and MDA-MB-231 breast cancer cells using Lipofectamine® RNAiMAX reagent (Invitrogen, cat# 13778-150). Briefly, 35 × 10^4^ MCF7 and 25 × 10^4^ MDA-MB-231 cells were seeded onto 6 cm dishes, and cultured overnight in antibiotic-free α-MEM and RPMI medium. 7.5 μL of survivin and XIAP siRNA oligomers (Cell Signaling Technology, cat# 6351 and cat# 6446) were diluted in 100 μL of Opti-MEM® I medium (Gibco, cat# 31985) without serum, and then mixed with 7.5 μL of Lipfectamine® RNAiMAX transfection reagent diluted in 98 μL Opti-MEM® I medium (Gibco) for 20 min at room temperature. Two microliters of HDAC 1, 2, 3, and 6 siRNA oligomers (Dharmacon, cat# M-003493-02-0005, cat# M-003495-02-0005, cat# M-003496-02-0005, and cat# M-003499-00-0005) were diluted in 98 μL of Opti-MEM® I medium (Gibco) without serum, and then mixed with 2 μL of Lipfectamine RNAiMAX transfection reagent diluted in 98 μL Opti-MEM® I medium (Gibco) without serum for 20 min at room temperature. Cells were overlaid with the transfection mixture, and incubated for 24–48 h.

### Western blot analysis

Cells were lysed in CelLytic^TM^ cell lysis reagent (Sigma, cat# C2978) containing 1 mM PMSF and 1 mM NaF with cocktail protease inhibitors (Roche, cat# 05892791001). Equal amounts of protein were subjected to SDS-PAGE on either an 8% or a 10% acrylamide gel. The resolved proteins were transferred to a PVDF membrane (Millipore), which was then exposed to 5% non-fat dried milk in TBST for 1 h at room temperature before incubation overnight at 4°C with primary antibodies. The PVDF membrane was then washed with TBS containing 0.05% Tween-20 before incubation for 1 h at room temperature with horse-radish peroxidase—conjugated goat antibodies to rabbit (Millipore, cat# AP132P), mouse (Millipore, cat# AP124P), or goat (Millipore, cat# AP106P) immunoglobulinG. Immunoreactive proteins were visualized using Western blot enhanced chemiluminescence reagents and protein signals were detected by luminescence readers (FUJI LAS-100, Tokyo, Japan). Experiments were repeated at least three times. Primary antibodies used in this study are listed as follows: mouse antibodies against β-actin and acetylated tubulin were obtained from Millipore (cat# MAB1501 and cat# 05829); antibodies against lamin A/C were from Santa Cruz (cat# sc-7292); antibodies against proteasome 26S and Hsp90 were from Abcam (cat# AB58115-100); and Enzo (cat# SPA-830), respectively. Goat antibodies against XIAP were obtained from Cell Signaling Technology (cat# 6466). Rabbit antibodies against LC3B were obtained from Origene (cat# TA301543); antibodies against survivin were from R&D system (cat# AF886); antibodies against acetylated survivin were from Novus (cat# NBP1-47639) and those against HDAC1, 2, 3, and 6 were from Gene Tex (cat# GTX100513, cat# GTX109642, cat# GTX109679, and cat# GTX100722).

### Reverse transcription and quantitative PCR (qPCR)

Total RNA was extracted using TRIzol® reagent (Invitrogen) and complementary DNA was synthesized from RNA using the RevertAid H Minus first strand cDNA synthesis kit (Thermo Scientific). Expression levels of survivin, XIAP, and actin transcript were determined by qPCR. The following primers were used in the study: human survivin forward primer, 5′-AGA ACTGGCCCTTCTTGGAGG; human survivin reverse primer, 5′-CTT TTTATGTTCCTCTATGGGGTC; human XIAP forward primer, 5′-CAA TATGGAGACTCAGCAGTTGGA; human XIAP reverse primer, 5′-GCA CTATTTTCAAGATAAAAGCCGTT; human beta-actin forward primer, 5′-GGC GGCACCACCATGTACCCT; human beta-actin reverse primer, 5′-AGG GGCCGGACTCGTCATACT; Experiments were repeated at least three times.

### Monodansylcadaverine (MDC) staining of acidic vesicular organelles (AVOs) and immunofluorescence staining of autophagosome/autophagolysosome

MDC staining was used to detect the formation of acidic vesicular organelles (AVOs) in breast cancer cells. Briefly, MCF7 and MDA-MB-231 cells were seeded on the 24-well plates and treated with 1/4x, 1/2x, 1x, 2x IC_50_ of SAHA or 1x IC_50_ of resveratrol for 72 h. AVOs were labeled with 0.5 mM MDC in the phenol red-free RPMI at 37°C for 2 h. Then, the cells were washed three times with PBS. To detect the formation of autophagosome or autophagolysosome in cells, immunofluorescence staining of LC3B was used. Briefly, MCF7 cells were seeded on glass coverslips. Cells were fixed with 4% paraformaldehyde for 15 min at room temperature and the fixed cells were then permeabilized with PBS containing 1% triton X-100 (Calbiochem) for 30 min, subsequently blocked in 5% bovine serum albumin (Sigma-Aldrich) for an hour and incubated with anti-LC3B primary antibodies overnight at 4°C. The cells were washed three times with TBS-Tween buffer, incubated with Alexa Fluor 594-conjugated secondary antibodies (Abcam, cat# ab150076) for an hour at room temperature, and suspended in blocking buffer containing DAPI for 15 min. AVOs and autophagosome/autophagolysosome in all cells were observed under a fluorescence microscope (Olympus, IX-71). Experiments were repeated at least three times.

### Protein stability assay

To measure the rate of protein degradation of survivin and XIAP, MCF7, and MDA-MB-231 cells were treated with 10 μg/mL of cycloheximide 72 h after SAHA treatment to inhibit *de novo* protein synthesis. Whole cell extracts were prepared from samples taken at 30 min time interval until 120 min, and the amounts of the survivin and XIAP protein present were determined by Western blotting. The rate of protein degradation was relative to the untreated control. Experiments were repeated three times.

### Proteasome activity assay

The proteasome activity assay was performed using a proteasome activity fluorometric assay kit (BioVision, cat# K245-100) according to the manufacturer's instructions. Briefly, proteasome inhibitor MG132 and proteasome substrates were added to the SAHA-treated cells. Samples were incubated in 37°C for 30 min. After incubation, the kinetics of the fluorescence development at Ex/Em = 350/440 nm were measured every 5 min for 30 min, and the luminescence was recorded on a SpectraMax® M5 microplate reader (Molecular Devices). The proteasome activity is calculated using the following equation: $Proteasome\;activity=\;\frac{B\;x\;sample\;dilution\;factor}{\left(T2-T1\right)x\;V}$.

### Statistical analysis

Each experiment was performed at least three times. Data are presented as mean (mean average of the replicated experiments) ± *s.e*. (standard error of the mean of the replicated experiments). The significance of difference was evaluated with one-way ANOVA. A value of *P* < 0.05 was considered to be statistically significant.

## Results

### SAHA induces autophagy and down-regulates the expression of survivin and XIAP in human breast cancer cells

**Figure 1:**
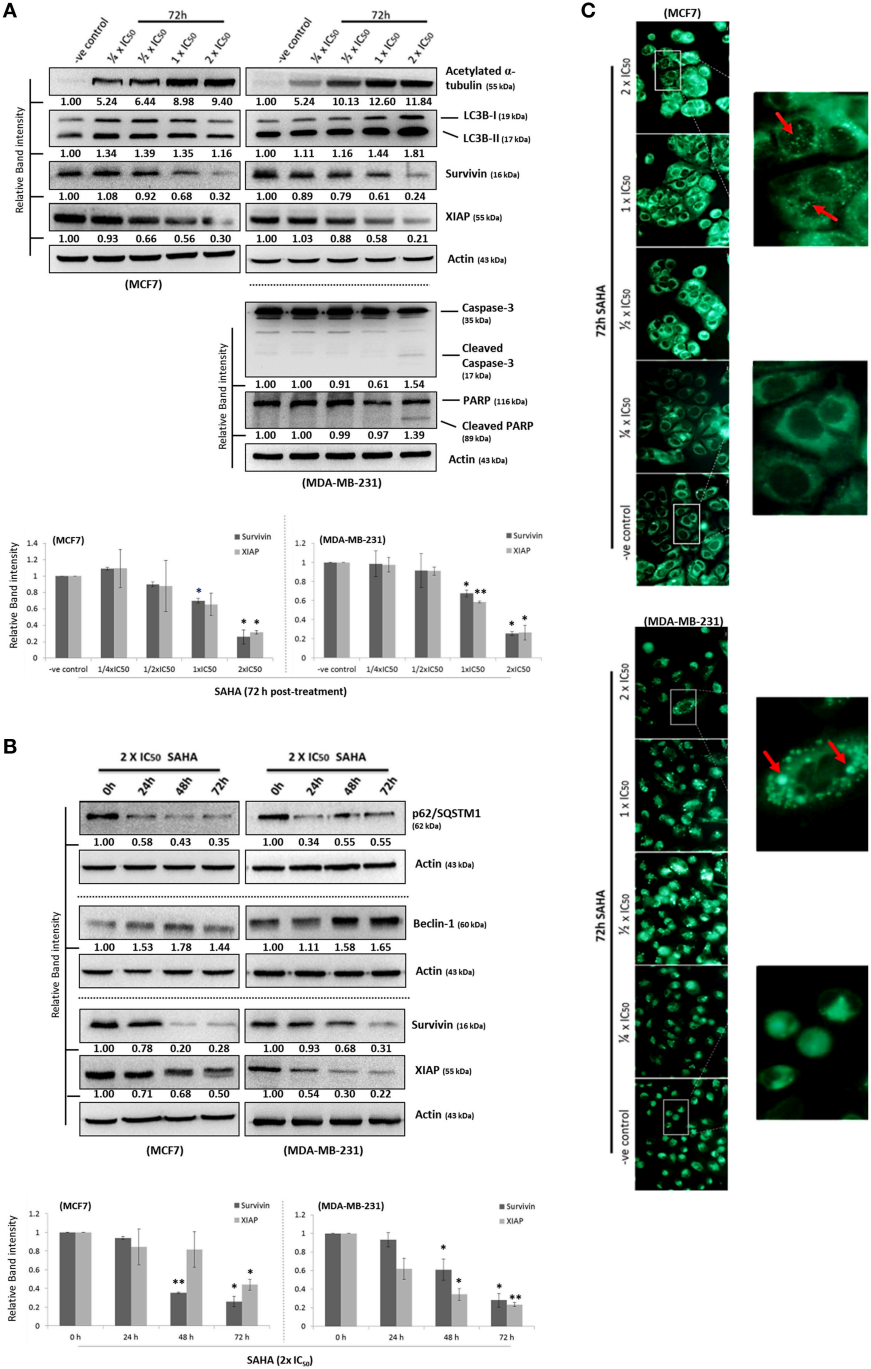
**SAHA concurrently induces autophagy and down-regulates the expression of survivin and XIAP in human breast cancer cells. (A,B)** MCF7 and MDA-MB-231 cells were treated with SAHA and expression of different proteins was analyzed by Western blotting. Equal protein loading was verified by actin. Experiments were repeated three times. The numbers under each blot are intensity of the blot relative to that of the untreated control (either “–ve control” or “0 h”). Signals in the survivin and XIAP blots (of all repeats) were quantitated and a graph was generated to show the effect of SAHA on the expression of survivin and XIAP. A statistically significant difference in the expression of survivin and XIAP in cells treated with SAHA vs. without SAHA (either “–ve control” or “0 h”) is denoted by “^*^” (*p* < 0.05) and “^**^” (*p* < 0.01). **(C)** MCF7 and MDA-B-231 breast cancer cells were treated with various concentrations of SAHA for 72 h and subsequently stained with MDC. AVOs in cells were observed under a fluorescence microscope. Red arrows indicate puncta formation.

To determine the effectiveness of SAHA in targeting various types of breast cancer *in vitro*, IC_50_-values for SAHA were determined. In this study, the ER^+^/caspase-3-deficient/p53-expressing MCF7 and its tamoxifen-resistant sub-lines (MCF7- TamR8 and MCF7-TamC3) were used. As shown in Table 1, MTT cell viability assay revealed that the IC_50_-values of SAHA in MCF7-TamC3 (0.9 ± 0.1 μM) and MCF7-TamR8 (1.2 ± 0.3 μM) were similar to that in the parental tamoxifen-sensitive MCF7 (0.7 ± 0.1 μM) cells. SAHA was also effective in targeting SK- BR-3 (ER^−^/HER2^+^, caspase-3, and p53 mutant-expressing) and the triple-negative MDA-MB-231 (ER^−^/HER2^−^/PR^−^, caspase-3, and p53 mutant-expressing) breast cancer cells at low micromolar concentrations (Table 1). Taken together, our results revealed that SAHA is effective in reducing cell viability of various breast cancer subtypes regardless of the expression and status of ER, HER2, caspase-3, and p53. Western blot analysis was used to investigate the molecular effects of SAHA in breast cancer cells. Two different breast cancer cell lines, MCF7 (ER^+^/tamoxifen-sensitive) and MDA-MB-231 (ER^−^/HER2^−^/PR^−^/tamoxifen-resistant), were selected for the following molecular investigations. As shown in Figure 1A, SAHA increased the expression of acetylated α-tubulin, which is a function indicator of the drug, in both MCF7 and MDA-MB-231 cells in a concentration-dependent manner. SAHA also increased the conversion of LC3B-II and expression of beclin-1, and decreased the expression of p62/SQSTM1, which are molecular markers for autophagy (Figures 1A,B). SAHA-treated cells were stained with monodansylcadaverine (MDC) to further determine the formation of acidic vesicular organelles (AVOs). MDC is a fluorescent compound commonly used for the detection of AVOs including lysosome and autolysosome (Niemann et al., 2000; Munafo and Colombo, 2001). Similar to the results of cancer cells treated with the known autophagy inducer, resveratrol, SAHA treatment also increased the formation of green fluorescent puncta, indicating the increased formation of AVOs (Supplementary Figure 1 and Figure 1C). Taken together, these results indicate that SAHA, at the tested concentrations, did function normally at the molecular level and induced autophagy in both MCF7 and MDA-MB-231 cells. Surprisingly, cleavage of caspase-3 and PARP, which are molecular markers for caspase-3 activation, was only observed in the pro-caspase-3 expressing MDA-MB-231 cells treated with high concentration (2x IC_50_) of SAHA (Figure 1A), suggesting that caspase-3 activation might only play a role in the cell viability reduction induced by SAHA at high concentrations but not in moderate-to-low concentrations.

**Figure 2:**
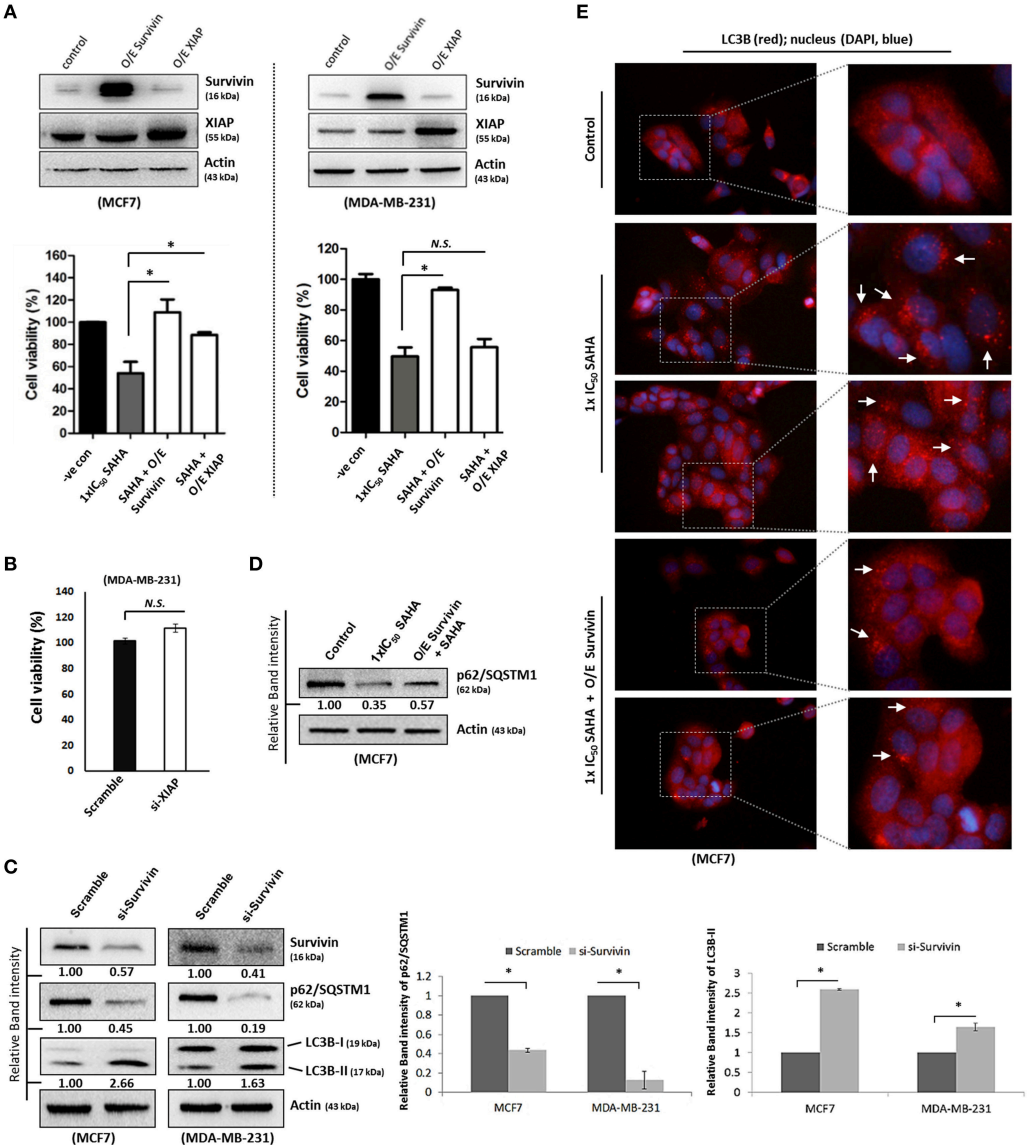
**Survivin plays an important role in SAHA-induced autophagy in breast cancer cells**. **(A**, upper panels) MCF7 and MDA-MB-231 cells were transfected with either pCMV-XL (control), pCMV-XL4-survivin [overexpresses (O/E) survivin] or pCMV-XL-5-XIAP (O/E XIAP) plasmid for 72 h. Expression of different proteins was analyzed by Western blotting. (**A**, lower panels) MCF7 and MDA-MB-231 cells were transfected with pCMV-XL (control), pCMV-XL4-survivin (O/E survivin) or pCMV-XL-5-XIAP (O/E XIAP) for 24 h prior to 72 h SAHA treatment. Cell viability was assessed by MTT assay. Experiment was repeated three times. A statistically significant difference in the viability of cells treated with O/E survivin or O/E XIAP + SAHA vs. SAHA alone is denoted by “^*^” (*p* < 0.05). “*N.S*.,” denotes no significant difference between the testing groups. **(B)** MDA-MB-231 cells were treated with either scramble siRNA or XIAP-specific siRNA (si-XIAP) for 72 h and cell viability was analyzed by MTT assay. **(C)** MCF7 cells were treated with either scramble siRNA or survivin-specific siRNA (si-Survivin) for 72 h. Expression of various proteins was analyzed by Western blotting. The numbers under each survivin blot are intensity of the blot relative to that of the scramble control. Signals in the p62/SQSTM1 blots (of all repeats) were quantitated and a graph was generated to show the effect of survivin on the expression of p62/SQSTM1. **(D)** Cells were transfected with pCMV-XL (control) for 72 h, pCMV-XL (control) for 24 h followed up with 48 h SAHA co-treatment, or pCMV-XL4-survivin (O/E survivin) for 24 h followed up with 48 h SAHA co-treatment. Expression of p62/SQSTM1 was analyzed by Western blotting. **(E)** Cells were transfected with pCMV-XL (control) for 72 h, pCMV-XL (control) for 24 h followed up with 48 h SAHA co-treatment, or pCMV-XL4-survivin (O/E survivin) for 24 h followed up with 48 h SAHA co-treatment. Formation of LC3B (red fluorescent) puncta in cells was observed under a fluorescence microscope and pointed out by the arrows in the photos. Nuclei were counterstained blue with DAPI.

Although survivin and its binding partner, XIAP, have been known for their anti-apoptotic functions in regulating cell survival and cell proliferation (Altieri, 2003; Potts et al., 2003), evidence that survivin and XIAP function as an endogenous repressor of autophagy has emerged (Wang et al., 2011; Huang et al., 2013; Cheng et al., 2015). SAHA is a class I HDAC (HDAC1, 2, and 3) and HDAC6 co-inhibitor and HDAC6 has been shown to regulate survivin expression through de-acetylation and cytoplasmic retention of survivin. Therefore, to determine whether SAHA-induced autophagy and/or decreased cell viability were at least partially caused by altering the expression of survivin and XIAP, the effects of SAHA on survivin and XIAP expression in both MCF7 and MDA-MB-231 cells were examined. Western blot analysis revealed that SAHA decreased the expression of survivin and XIAP in both concentration- and time-dependent manners (Figures 1A,B). Then, we evaluated the impact of the expression of survivin and XIAP on the effectiveness of SAHA in reducing cell viability and up-regulating autophagy in breast cancer cells. MTT assay revealed that ectopic over-expression of survivin significantly attenuated the inhibitory effect of SAHA (at 1x IC_50_ conc.) on cell viability in both MCF7 and MDA-MB-231 cells (Figure 2A). Surprisingly, ectopic over-expression of XIAP only attenuated the cell viability inhibitory effect of SAHA in MCF7 but not in MDA-MB-231 breast cancer cells (Figure 2A). In addition, down-regulation of XIAP alone by siRNA did not affect the viability of MDA-MB-231 cells *in vitro* (Figure 2B). These results indicated that XIAP might only play a minor role and/or a cell line-dependent role in SAHA-induced cell viability reduction, whereas, survivin might play a major role in facilitating SAHA induced cell viability reduction in human breast cancer cells.

Because down-regulation of survivin by siRNA induced autophagy as indicated by the decreased expression of p62/SQSTM1 and increased conversion of LC3B-II in both MCF7 and MDA-MB-231 cells (Figure 2C), experiments were carried out to determine whether survivin down-regulation plays a role in SAHA-induced autophagy, which contributes to the subsequent cell death. Ectopic over-expression of survivin was performed and changes in the expression of p62/SQSTM1 and the formation of autophagosome in the SAHA-treated MCF7 cells was analyzed by Western blotting and immunofluorescence microscopy. As shown in Figure 2D, SAHA treatment (at 1x IC_50_ conc.) decreased the expression of p62/SQSTM1, which is an autophagic flux marker, in MCF7 cells as expected. SAHA treatment also increased the formation of LC3B (red fluorescent) puncta, indicating increased formation of autophagosome/autophagolysosome in cells (Figure 2E). Noticeably, ectopic over-expression of survivin partially restored the expression of p62/SQSTM1 and attenuated the effect of SAHA on LC3B puncta formation in the SAHA-treated cells (Figures 2D,E). Taken together, these results indicate that SAHA upregulates autophagy partially through down-regulation of survivin.

### SAHA decreases the amount of survivin and XIAP mRNA transcripts present in breast cancer cells

**Figure 3:**
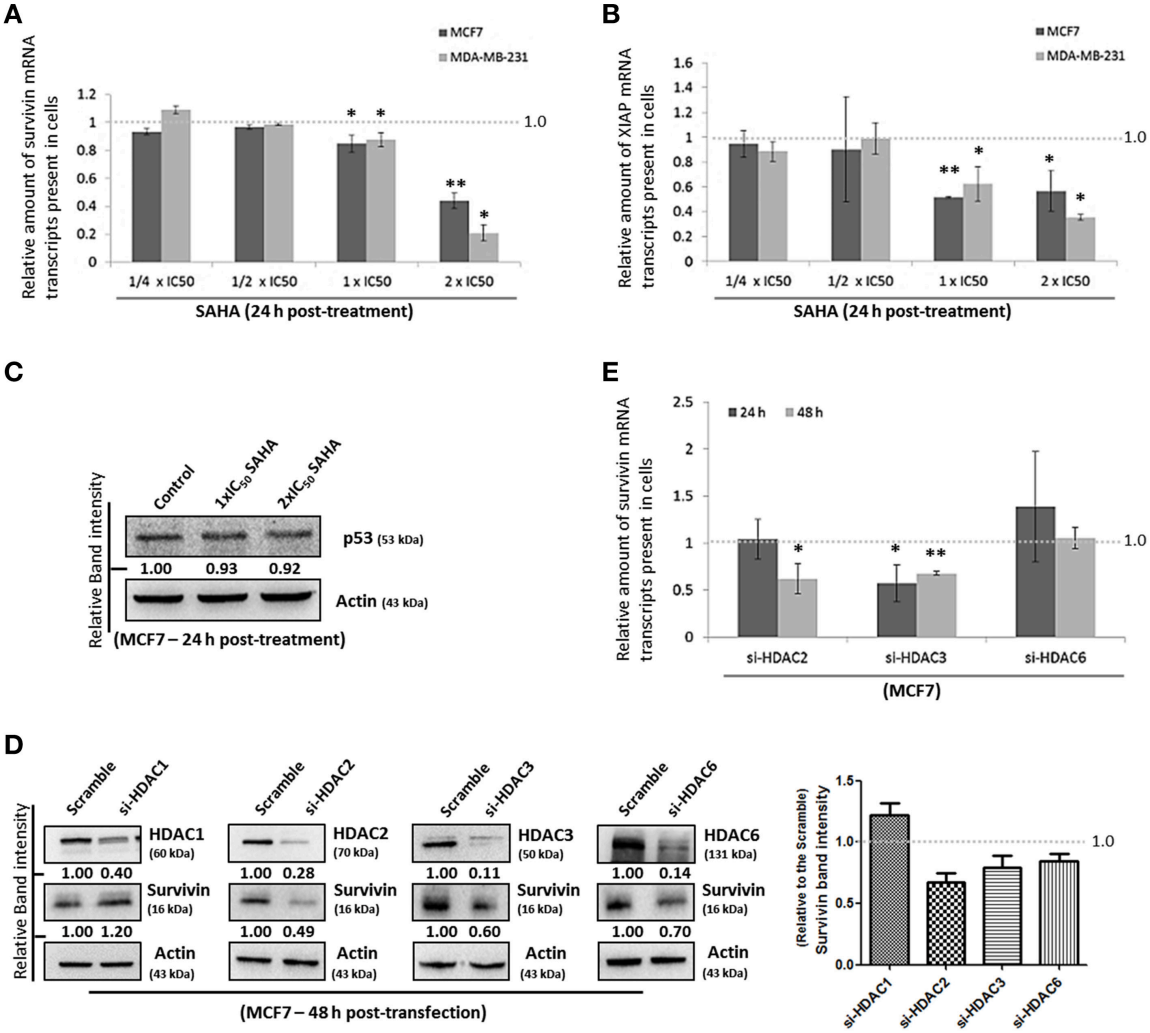
**SAHA affects the expression of suvivin and XIAP at the transcriptional level**. **(A)** MCF7 and **(B)** MDA-MB-231 cells were treated with various concentrations of SAHA for 24 h. The relative amount of survivin and XIAP mRNA transcripts present in cells was analyzed by qPCR. Experiment was repeated three times. A statistically significant difference in the amount of mRNA transcripts present in cells treated with SAHA vs. without SAHA (negative control) is denoted by “^*^” (*p* < 0.05) and “^**^” (*p* < 0.01). **(C)** MCF7 cells were treated with SAHA for 24 h and expression of p53 was determined by Western blotting. **(D)** MCF7 cells were transfected with HDAC1, 2, 3, or 6 siRNA for 48 h. Expression of different proteins was analyzed by Western blotting. Signals in the survivin blots (of all repeats) were quantitated and a graph was generated to show the effect of different HDACs on the expression of survivin. **(E)** MCF7 cells were transfected with scramble, HDAC2, 3, or 6 siRNA for either 24 or 48 h. Relative amount of survivin mRNA transcripts present in cells was determined by qPCR. Experiment was repeated three times. A statistically significant difference in the amount of mRNA transcripts present in cells treated with HDAC2, 3, or 6 siRNA vs. scramble siRNA is denoted by either “^*^” (*p* < 0.05) or “^**^” (*p* < 0.01).

Next, we sought to investigate which pathway could SAHA be involved in suppressing the expression of survivin and XIAP; we examined the effects of SAHA on survivin and XIAP at the transcriptional level. Quantitative real-time PCR analysis revealed that SAHA (24 h post-treatment, at 1–2x IC_50_ conc.) decreased the amount of survivin and XIAP mRNA transcripts present by approximately 50% in both MCF7 and MDA-MB-231 cells (Figures 3A,B), indicating that SAHA, at least at the tested concentrations, can reduce the expression of survivin and XIAP at the transcriptional level. In addition, Western blot analysis revealed that SAHA did not affect the expression of p53, which is a survivin gene transcription negative regulator, in the wild-type p53 expressing MCF7 cells, further indicating that SAHA exhibits its anti-breast cancer effect, at least at the transcriptional level, through a p53-independent mechanism (Figure 3C; Mirza et al., 2002).

To identify which isoforms of HDACs are the major contributors in SAHA-mediated survivin depletion at the transcriptional level, we treated the cells with HDAC1, HDAC2, HDAC3, and HDAC6 specific siRNA. Results of the Western blot analysis revealed that down-regulation of HDAC2, HDAC3, and HDAC6 decreased the expression of survivin, whereas down-regulation of HDAC1 increased the expression of survivin in MCF7 cells (Figure 3D). Down-regulation of HDAC1 by siRNA also increased the expression of survivin in MDA-MB-231 cells (Supplementary Figure 2A). Therefore, quantitative real-time PCR was performed to determine whether HDAC2, HDAC3, and HDAC6 (but not HDAC1) can regulate survivin gene expression in breast cancer cells. Down-regulation of HDAC6 by siRNA did not significantly alter the amount of survivin mRNA transcripts present in MCF7 cells, whereas, down-regulation of HDAC2 and HDAC3 significantly decreased the amount of survivin mRNA transcripts present in cells (Figure 3E). Collectively, these results indicate that SAHA might down-regulate survivin expression at the transcriptional level in part through HDAC2 and HDAC3 co-inhibitions.

### SAHA promotes the degradation of survivin protein in human breast cancer cells in part through survivin acetylation and nuclear translocation

**Figure 4:**
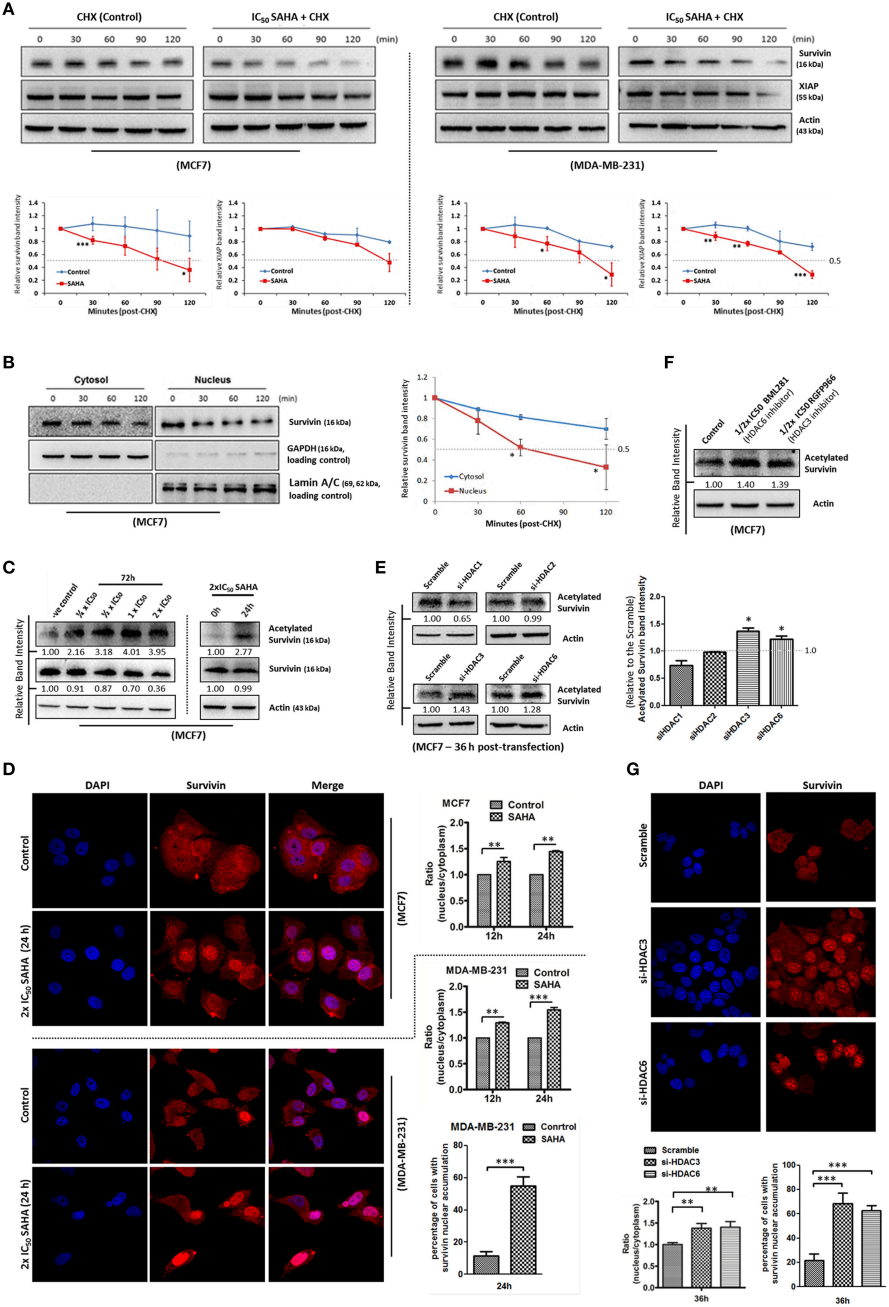
**SAHA decreases the protein stability of survivin and XIAP in breast cancer cells. (A)** Breast cancer cells were treated with 1x IC_50_ SAHA for 72 h. Cycloheximide (CHX) was added 72 h post-SAHA treatment to the cells to inhibit *de novo* protein synthesis. Cells were then harvested at the time points indicated and expression of survivin and XIAP was analyzed by Western blotting. Experiments were repeated three times and representative blots were shown. Signals in the blots (of all repeats) were quantitated and a graph was generated to compare the degradation rates. A statistically significant difference in the mean of the relative band intensity (of all repeats) of survivin and XIAP in cells treated with SAHA vs. without SAHA (control) at the same time point is denoted by “^*^” (*p* < 0.05), “^**^” (*p* < 0.01), or “^***^” (*p* < 0.001). **(B)** MCF7 cells were treated with cycloheximide to inhibit the *de novo* protein synthesis process. Nucleic proteins and cytoplasmic proteins were isolated using cells fractionation assay. Expression of survivin was analyzed by Western blotting. Equal protein loading was verified by either lamin A/C or GAPDH. The numbers under each blot are intensity of the blot relative to that of the control (0 h). Signals in the blots (of all repeats) were quantitated and a graph was generated to compare the degradation rates. A statistically significant difference in the mean of the relative band intensity (of all repeats) of nuclear survivin and cytosolic survivin is denoted by “^*^” (*p* < 0.05). **(C)** MCF7 cells were treated with SAHA and expression of the acetylated survivin was analyzed by Western blotting. **(D)** Breast cancer cells were treated with 2x IC_50_ SAHA for 24 h. Intracellular distribution of survivin was analyzed using immunofluorescence confocal microscopy. Nucleus was stained blue with DAPI. Survivin was labeled red in the photo. Relative expression of nucleic and cytoplasmic survivin in cells treated with/without SAHA was quantified. Experiment was repeated three times. A statistically significant difference in the nucleus/cytoplasm ratio of red fluorescence (survivin) intensity in cells with SAHA vs. without SAHA (control) is denoted by either “^**^” (*p* < 0.01) or “^***^” (*p* < 0.001). Percentage of cells with red fluorescence signal higher in the nucleus than in the cytosol in cells treated with or without SAHA was also quantified. **(E)** MCF7 cells were transfected with HDAC1, 2, 3, or 6 siRNA for 36 h. Expression of acetylated survivin was determined by Western blotting. Signals in the acetylated survivin blots (of all repeats) were quantitated and a graph was generated to show the effect of different HDAC isoforms on the expression of acetylated survivin. A statistically significant difference in the expression of acetylated survivin in cells treated with HDAC1, 2, 3, or 6 siRNA vs. scramble siRNA is denoted by “^*^” (*p* < 0.05). **(F)** MCF7 cells were treated with or without BML281 and RGFP966 for 48 h. Expression of acetylated survivin was determined Western blotting. **(G)** MCF7 cells were transfected with scramble, HDAC3 or HDAC6 siRNA for 36 h. Intracellular distribution of survivin was analyzed using immunofluorescence confocal microscopy. Nucleus was stained blue with DAPI. Survivin was labeled red in the photo. Relative expression of nucleic and cytoplasmic survivin in cells treated with scramble, HDAC3 or HDAC6 siRNA was quantified. Experiment was repeated three times. A statistically significant difference in the nucleus/cytoplasm ratio of red fluorescence (survivin) intensity in cells treated with HDAC3 or 6 siRNA vs. scramble siRNA is denoted by “^**^” (*p* < 0.01). Percentage of cells with red fluorescence signal higher in the nucleus than in the cytosol in cells treated with or without HDAC3 and HDAC6 siRNA was also quantified.

To determine whether SAHA also affects the expression of survivin and XIAP through other mechanisms, the protein stability of both survivin and XIAP was evaluated. Western blot analysis revealed that the protein stability of survivin was significantly decreased in both MCF7 and MDA-MB-231 cells treated with SAHA (at IC_50_ conc.) relative to the control (Figure 4A). The protein stability of XIAP was also significantly decreased in SAHA-treated MDA-MB-231 cells and slightly decreased in SAHA-treated MCF7 cells as compared to that of the untreated cells (Figure 4A).

Survivin shuttles between the nucleus and the cytoplasm through active nucleocytoplasmic transport (Connell et al., 2008). Among different HDAC isoforms, HDAC6 has been shown to play a role in the de-acetylation and cytoplasmic retention of survivin in cancer cells. Acetylation of the lysine 129 (K129) residue of survivin promotes nuclear translocation of survivin, and nuclear survivin has been shown to exhibit reduced protein stability as compare to the cytosolic survivin (Wang et al., 2010). Therefore, we sought to determine whether SAHA down-regulates survivin expression partially through survivin acetylation and nuclear translocation. Here, results from the protein stability assay by treating MCF7 with the *de novo* protein synthesis inhibitor, cycloheximide, revealed that the stability of the nuclear survivin protein was lower than that of the cytosolic survivin protein, which is consistent with the findings of previous studies (Figure 4B). Western blot analysis also revealed that SAHA promoted the acetylation of survivin in MCF7 cells in a concentration-dependent manner (Figure 4C). To further confirm that SAHA treatment did induce survivin nuclear translocation in the treated breast cancer cells, immunofluorescence microscopy was performed. Results from the immunofluorescence microscopy showed that SAHA promoted survivin nuclear accumulation in both MCF7 and MDA-MB-231 cells at 12–24 h post-treatment (Figure 4D). To identify which isoforms of HDAC are the major contributors in SAHA-mediated survivin acetylation and nuclear translocation, we treated the cells with HDAC1, HDAC2, HDAC3, and HDAC6 specific siRNA. Here, results of the Western blot analysis showed that down-regulation of HDAC6 induced survivin acetylation as expected (Figure 4E). Interestingly, down-regulation of HDAC3 also induced survivin acetylation in MCF7 cells (Figure 4E). To confirm that HDAC6 and HDAC3 could regulate survivin acetylation in breast cancer cells, MCF7 cells were treated with BML281 and RGFP966, which are pharmacological inhibitors of HDAC6 and HDAC3, respectively, and the expression of acetylated survivin was again determined by Western blotting. Similar to the results of cells treated with HDAC6 siRNA and HDAC3 siRNA, inhibiting HDAC6 and HDAC3 by pharmacological inhibitors also increased the expression of acetylated survivin in MCF7 cells (Figure 4F). Immunofluorescence microscopy was performed to confirm the promotion of nuclear translocation of survivin in cells with HDAC3 and HDAC6 down-regulations. Here, down-regulation of HDAC6 and HDAC3 by siRNA clearly induced survivin nuclear accumulation in MCF7 cells (Figure 4G). Taken together, these results indicate that SAHA decreases survivin protein stability at least partially through HDAC3 and HDAC6 regulated survivin acetylation and nuclear translocation.

### SAHA increases 26S proteasome expression and decreases Hsp90 expression in breast cancer cells

**Figure 5:**
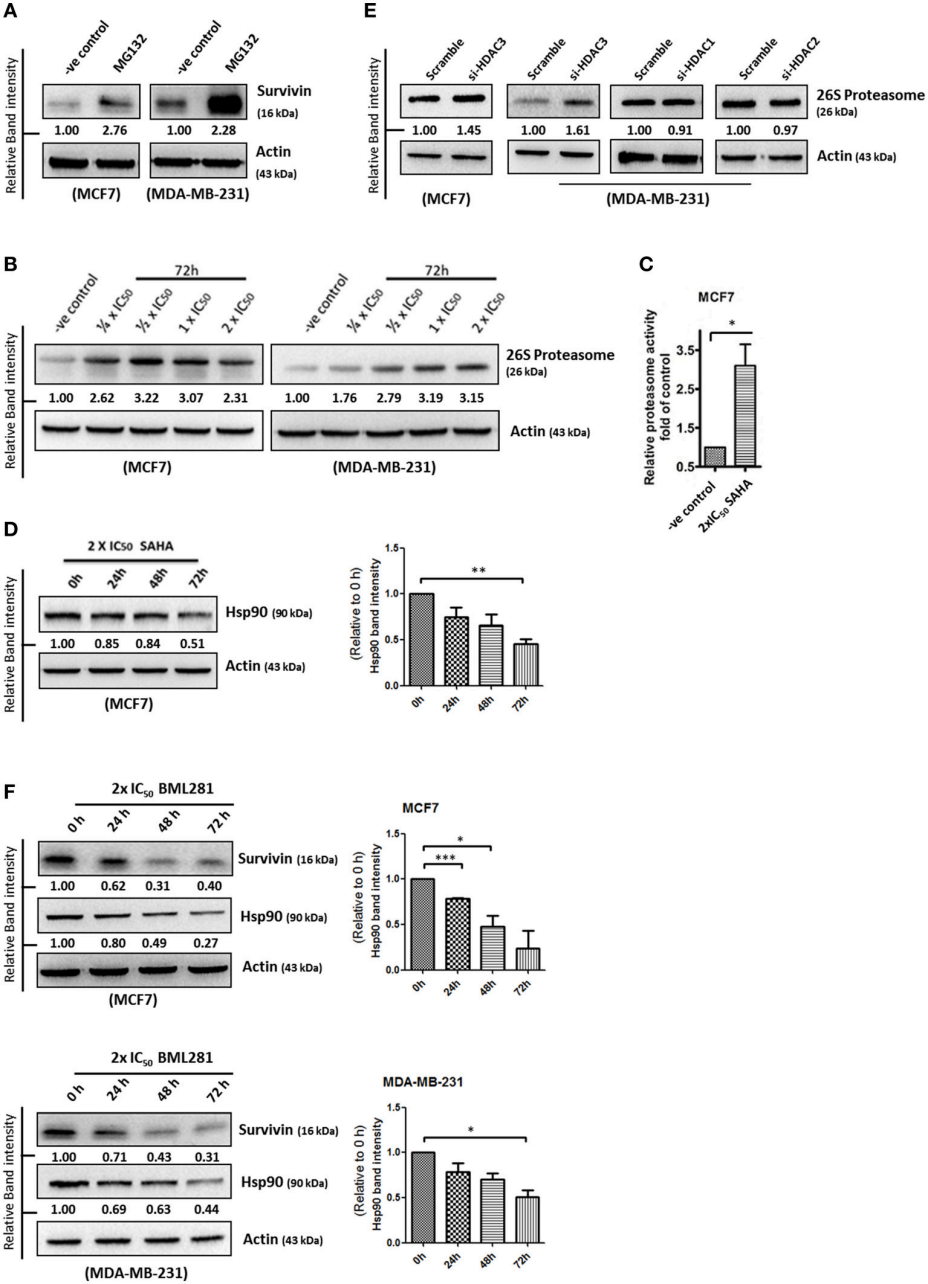
**SAHA increases the expression of 26S proteasome and decreases the expression of Hsp90 in breast cancer cells. (A)** Breast cancer cells were treated with the pharmacological inhibitor of proteasome, MG132, for 48 h. Expression of survivin was determined by Western blotting. **(B)** Breast cancer cells were treated with indicated concentrations of SAHA for 72 h and the expression of 26S proteasome was analyzed by Western blotting. **(C)** Breast cancer cells were treated with SAHA for 72 h and the intracellular proteasome activity in the treated cells were assessed using proteasome activity fluorometric assay kit. Experiment was repeated three times. A statistically significant difference in the proteasome activity in cells treated with SAHA vs. without SAHA (control) is denoted by “^*^” (*p* < 0.05). **(D)** MCF7 cells were treated with SAHA for indicated durations and expression of Hsp90 was determined by Western blotting. Signals in the Hsp90 blots (of all repeats) were quantitated and a graph was generated to show the effect of SAHA on the expression of Hsp90. A statistically significant difference in the expression of Hsp90 in cells treated with SAHA vs. without SAHA (0 h) is denoted by “^**^” (*p* < 0.01). **(E)** Breast cancer cells were transfected with scramble, HDAC3, HDAC1, or HDAC2 siRNA for 72 h. Expression of various proteins was determined by Western blotting. **(F)** Breast cancer cells were treated with BML281 for 24–72 h. Expression of different proteins was determined by Western blotting. Signals in the Hsp90 blots (of all repeats) were quantitated and a graph was generated to show the effect of BML281 on the expression of Hsp90. A statistically significant difference in the expression of Hsp90 in cells treated with BML281 vs. without BML281 (0 h) is denoted by either “^*^” (*p* < 0.01) or “^***^” (*p* < 0.001).

Survivin expression is also post-translationally regulated by the proteasomal protein degradation pathway. Binding of Hsp90 prevents survivin undergoing ubiquitination and the subsequent protein degradation by proteasome (Fortugno et al., 2003). To determine whether SAHA affects the cellular proteasomal protein degradation pathway, leading to the reduction of survivin protein stability in breast cancer cells, the expression of 26S proteasome and Hsp90 in SAHA treated breast cancer cells was determined. Western blot analysis was first performed to confirm the importance of proteasome in regulating survivin expression in breast cancer cells. Here, inhibiting proteasome by MG132 clearly increased survivin expression in both MCF7 and MDA-MB-231 cells, confirming that survivin expression is regulated post-translationally by proteasome (Figure 5A). Western blot analysis and proteasome activity assay revealed that SAHA treatment increased both the expression and activity of 26S proteasome in breast cancer cells (Figures 5B,C). In contrast, the same treatment decreased the expression of Hsp90 in MCF7 cells (Figure 5D). Interestingly, down-regulation of HDAC3, but not other HDAC isoforms, by siRNA increased the expression of 26S proteasome in breast cancer cells (Figure 5E). In addition, similar to the results of cells treated with SAHA, inhibiting HDAC6 by BML281 also down-regulated the expression of Hsp90 in both MCF7 and MDA-MB-231 cells, indicating that SAHA might down-regulate Hsp90 expression through HDAC6 inhibition (Figure 5F). Collectively, these results indicated that SAHA might promote survivin protein degradation in part through decreasing the ubiquitination protection from Hsp90 *via* HDAC6 inhibition and increasing the expression of 26S proteasome *via* HDAC3 inhibition in breast cancer cells.

## Discussion

Autophagy is a double-edged sword. It promotes cell survival in cells under genotoxic stress, metabolic stress, and energy starvation (Ogata et al., 2006; Qiang et al., 2013). However, excessive or prolonged autophagy may reduce cell viability by promoting autophagic cell death (Baehrecke, 2005; Szlosarek, 2014; Wang et al., 2015). Several studies have shown that SAHA induces autophagy, apoptosis, and exhibits potent anti-proliferative activity in cancer cells; however, the exact mechanisms by which SAHA induces these effects have not been fully understood (Butler et al., 2002; Lee et al., 2012). Survivin and XIAP are members of the IAPs and traditionally, these molecules are only believed to play important roles in regulating mitosis and inhibiting apoptosis. Therefore, it is seldom thought that survivin and XIAP play a role in SAHA-induced autophagy in cancer cells. Our previous study revealed that targeting survivin by its pharmacological inhibitor, YM155, induces autophagy and autophagic cell death without caspase-3 activation in breast cancer cells, indicating that: (1) caspase-3 activation is not a definite prerequisite event for YM155 induced breast cancer cell death and (2) survivin might play a role in the regulation of cellular autophagy (Cheng et al., 2015). In this study, we found that SAHA is equally potent toward the caspase-3 deficient MCF7 cells and its tamoxifen-resistant sublines, and the caspase-3 expressing MDA-MB-231 cells *in vitro*. We also found that SAHA down-regulated survivin and XIAP expression in both MCF7 and MDA-MB-231 breast cancer cells. Importantly, we demonstrated that ectopic expression of survivin completely attenuated the effect of SAHA on cell viability reduction and also partially attenuated the effect of SAHA on autophagy induction.

The role of XIAP in SAHA-induced autophagy and SAHA-reduced cell viability in cancer cells remains controversial. In this study, SAHA clearly induced XIAP down-regulation in the tested cell lines. However, ectopic over-expression of XIAP attenuated the effect of SAHA only in MCF7 but not in MDA-MB-231 cells. Interestingly, down-regulation of XIAP alone by siRNA did not affect the viability of MDA-MB-231 cells. In fact, Sensintaffar et al. also demonstrated that down-regulation of XIAP was ineffective in reducing the cell viability of various cancer cell lines including MCF7, HCT116 (colon), and PC3 (prostate) *in vitro* (Sensintaffar et al., 2010). It is also worth noting that down-regulation of HDAC2, 3, and 6 by siRNA all decreased survivin expression and concurrently increased LC3B-II conversion in MCF7 and MDA-MB-231 cells (Supplementary Figure 2B). In contrast, down-regulation of HDAC1 decreased XIAP expression in MCF7 and MDA-MB-231 cells, but the same treatment increased survivin expression and concurrently decreased LC3B-II conversion in cells (Supplementary Figures 2A,B). Taken together, it seems that induction of autophagy, at least as indirectly indicated by the increased conversion of LC3B-II, only occurred in breast cancer cells with survivin down-regulation but not with XIAP down-regulation following the knockdown of different HDAC isoforms by siRNA. Therefore, unlike survivin, XIAP may only play a minor and also a cell line-dependent role in SAHA-induced autophagy and cell viability reduction in breast cancer cells.

**Figure 6:**
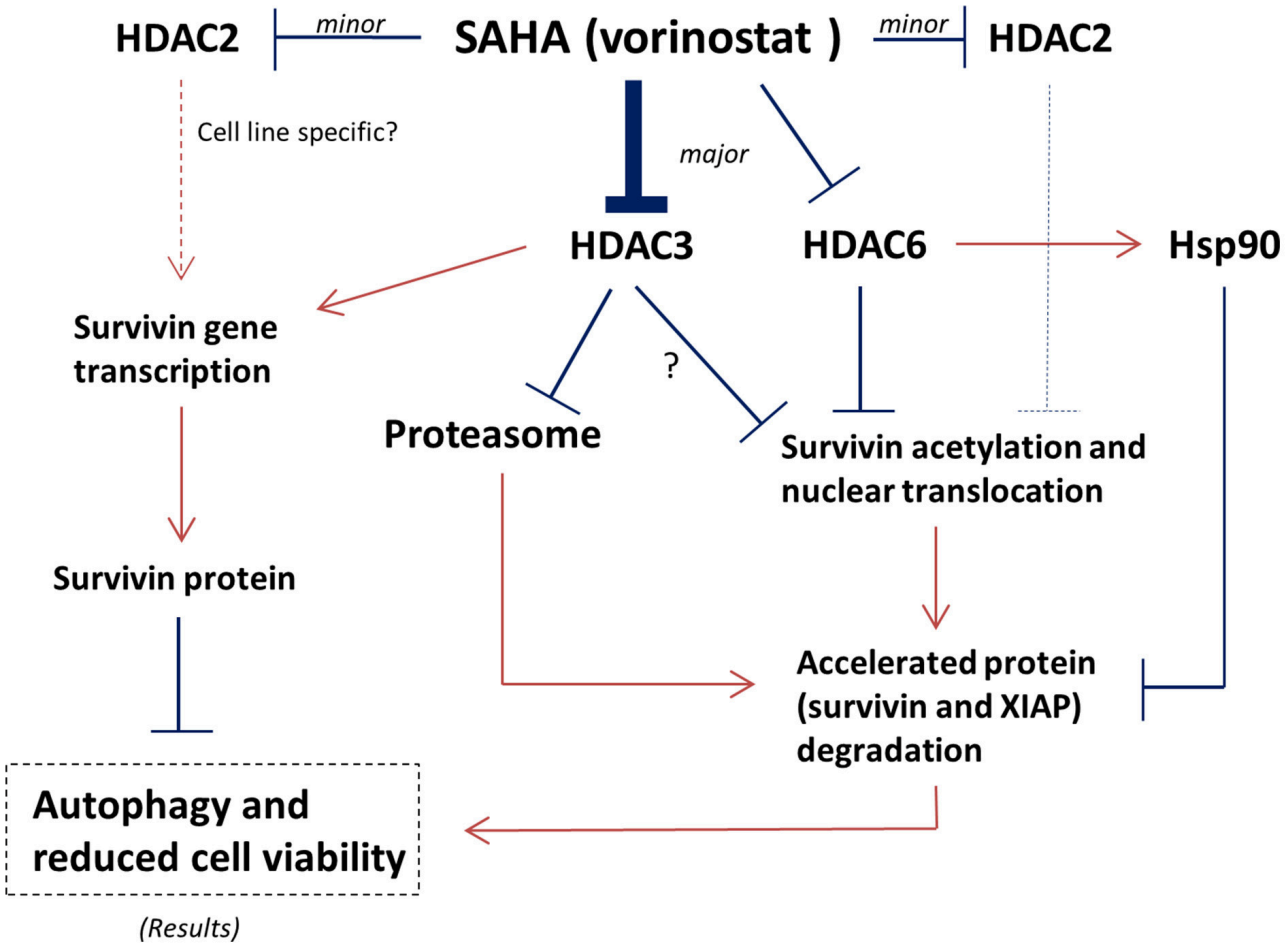
**Proposed mechanisms of SAHA in the expression regulation of survivin in human breast cancer cells**. Arrow indicates activation; Bar indicates inhibition.

Although SAHA is a class I HDAC and HDAC6 co-inhibitor, SAHA still exerts selectivity toward different HDAC isoforms. Through *in vitro* HDAC activity assay, SAHA was reported to be more effective in targeting HDAC6 (IC_50_ = 0.009 μM) and HDAC3 (IC_50_ = 0.019 μM; Hanson et al., 2013). In addition, targeting either HDAC3 or HDAC6 could also decrease the expression of survivin and increased the conversion of LC3-B-II in mutant p53-expressing SK-BR-3 breast cancer cells, further supporting the role of HDAC3 and HDAC6 in regulating survivin expression in the SAHA treated breast cancer cells regardless to the p53 status (Supplementary Figure 3). It has been shown that HDAC6 plays an important role in survivin deacetylation and the subsequent nuclear export of survivin (Riolo et al., 2012). Therefore, it is not surprising to see that SAHA induced survivin acetylation and nuclear accumulation in MCF7 and MDA-MB-231 breast cancer cells in this study, given that SAHA is most potent in targeting HDAC6. However, it is interesting to see that down-regulation of HDAC3 (the second most selected target of SAHA) by siRNA also induced survivin acetylation and nuclear translocation in breast cancer cells. In fact, regulation of survivin acetylation and nuclear translocation by HDAC isoform other than HDAC6 has not been shown in the past. Furthermore, down-regulation of HDAC3 by siRNA also mimicked the effect of SAHA in modulating survivin expression at the transcriptional level in MCF7 cells. A study by Jung et al. showed that 1-stearoyl-sn-glycero-3-phosphocholine (LPC), one of the lysophosphatidylcholines, decreased HDAC3 expression and suppressed the binding of HDAC3 to the promotor of survivin in chronic myelogenous leukemia K562 cells *in vitro* (Jung et al., 2013). Therefore, it is possible that inhibition of HDAC6 and HDAC3 all contributed to the SAHA induced survivin acetylation, nuclear translocation, and the subsequent protein degradation in breast cancer cells. Inhibition of HDAC3 further contributed to the SAHA induced gene transcription reduction of survivin in the treated cells (Figure 6).

Besides inducing the acetylation and nuclear translocation of survivin protein, SAHA also increased the expression of 26S proteasome in the tested cell lines. It is well-known that survivin associates with Hsp90 *via* an interaction that involves the ATPase domain of Hsp90. Binding of Hsp90 prevents survivin undergoing ubiquitination and the subsequent protein degradation by proteasome (Fortugno et al., 2003). Intriguingly, inactivation of HDAC6 has been shown to induce Hsp90 hyperacetylation, resulting in a loss of chaperone activity (Kovacs et al., 2005). In our study, inhibiting HDAC6 decreased the expression of Hsp90 in MCF7 and MDA-MB-231 cells. Therefore, SAHA might down-regulate survivin expression through transcriptional modulation and multiple post-translational mechanisms including increases in the rate of proteasomal protein degradation, the amount of protein acetylation, and the level of nuclear translocation *via* HDAC6 and HDAC3 inhibitions (Figure 6).

In conclusion, our study reveals that SAHA has potential for the management of various breast cancer subtypes regardless of the expression of ER and tamoxifen sensitivity. Our study also reveals that down-regulation of survivin gene transcription and protein stability by the inhibition of HDAC6 and HDAC3 might play important roles in both SAHA-induced autophagy and SAHA-reduced cell viability in breast cancer cells. Our findings emphasize the complexity of the regulatory roles in different HDAC isoforms and potentially assist in predicting the mechanism of novel HDAC inhibitors in targeted or combinational therapies in the future (Shi et al., 2010).

## Author contributions

Conceived and designed the experiments: JL and CHC. Performed the experiments: JL, HC, CK, SHC, CHL, KL, SMC, ST, and CFL. Analyzed the data: JL, EL, WH, YW, and CHC. Wrote and proofread the paper: JL, EL, JK, CCC, and CHC.

## Funding

This work is kindly supported by the following grants: NSC102-2320-B-006-038, MOST 104-2320-B-006-029 (Ministry of Science and Technology, Taiwan R.O.C) and NHRI-EX103-10237SC, NHRI-EX104-10237SC (National Health Research Institutes, Taiwan R.O.C).

### Conflict of interest statement

The authors declare that the research was conducted in the absence of any commercial or financial relationships that could be construed as a potential conflict of interest.

## Supplementary material

Supplementary Figure 1 **Resveratrol induces the formation of AVOs in breast cancer cells**. MCF7 and MDA-B-231 breast cancer cells were treated with 1x IC_50_ resveratrol for 72 h and subsequently stained with MDC. AVOs in cells were observed under a fluorescence microscope. Red arrows indicate green fluorescence puncta formation.

Supplementary Figure 2 **Down-regulation of different HDAC isoforms induces differential effects on the expression of survivin and conversion of LC3B-II in breast cancer cells. (A)** MDA-MB-231 and **(B)** MCF7 cells were transfected with either scramble siRNA or different HDAC isoforms specific siRNA for 48 h. Expression of different proteins and conversion of LC3B-II were determined by Western blotting.

Supplementary Figure 3 **Inhibiting HDAC3 and HDAC6 decreases survivin expression and increases LC3B-II conversion in Sk-Br-3 cells**. Sk-Br-3 breast cancer cells were treated with either RGFP966 or BML281 and the expression of various proteins was determined by Western blotting.
