# Supplementary figures and images for: Inhibition of HDAC3- and HDAC6-Promoted Survivin Expression Plays an Important Role in SAHA-Induced Autophagy and Viability Reduction in Breast Cancer Cells
Source: Front Pharmacol. 2016 Mar 31;7:81. doi: 10.3389/fphar.2016.00081 (PMC4814469; doi:10.3389/fphar.2016.00081)

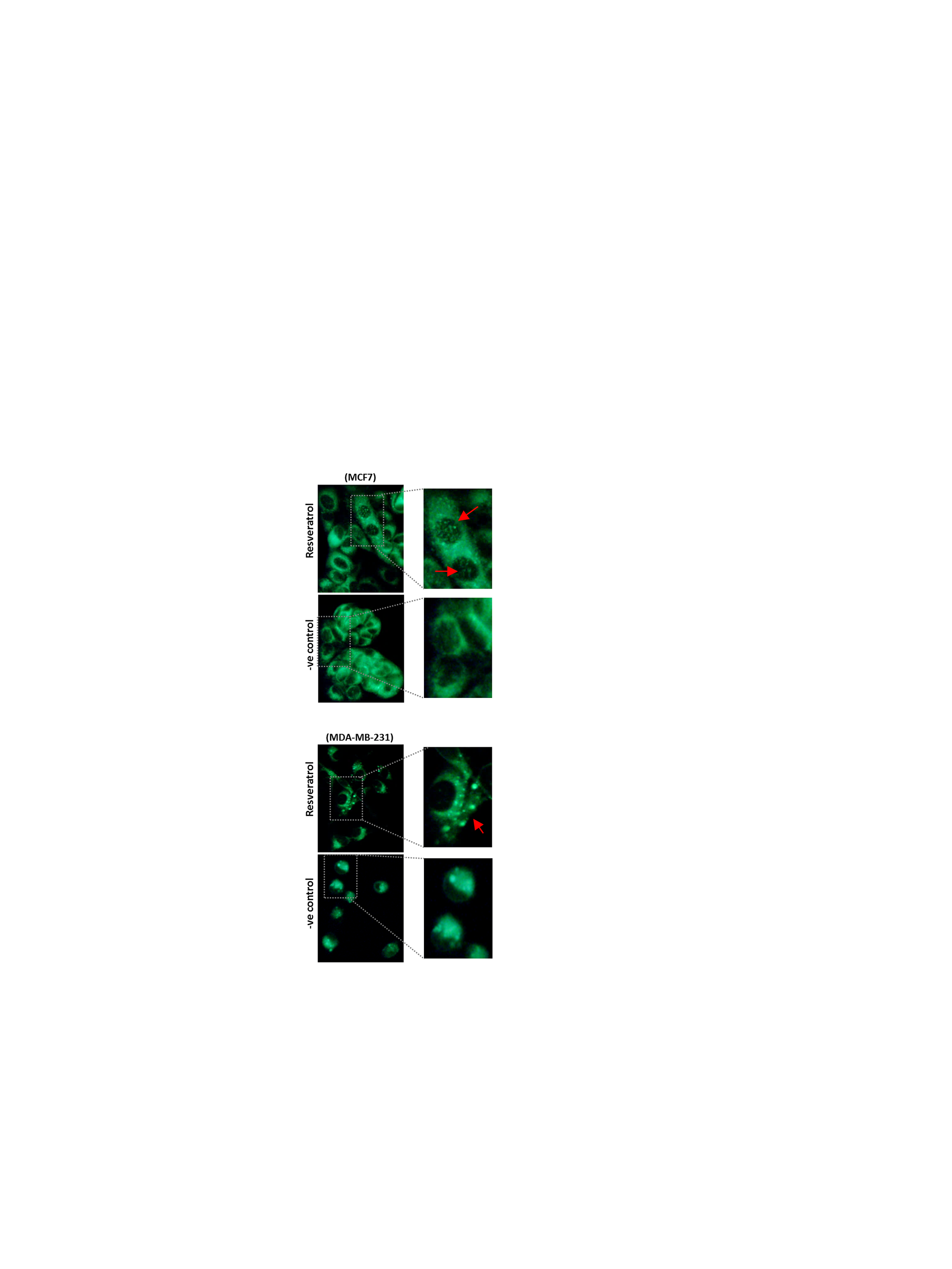

Supplement: Supplementary Figure 1 — Resveratrol induces the formation of AVOs in breast cancer cells. MCF7 and MDA-B-231 breast cancer cells were treated with 1x IC50 resveratrol for 72 h and subsequently stained with MDC. AVOs in cells were observed under a fluorescence microscope. Red arrows indicate green fluorescence puncta formation. [file Image1.TIF]

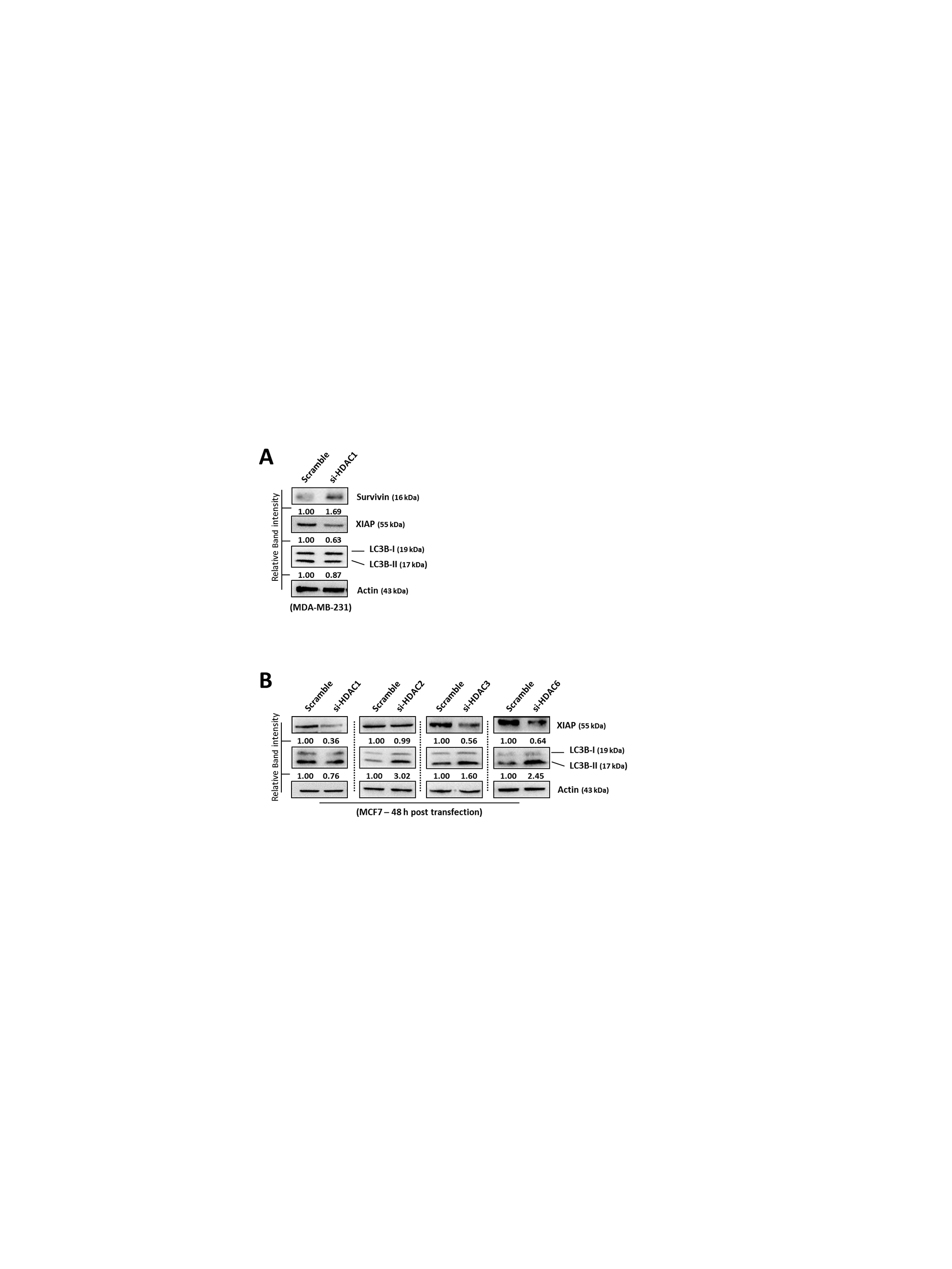

Supplement: Supplementary Figure 2 — Down-regulation of different HDAC isoforms induces differential effects on the expression of survivin and conversion of LC3B-II in breast cancer cells. (A) MDA-MB-231 and (B) MCF7 cells were transfected with either scramble siRNA or different HDAC isoforms specific siRNA for 48 h. Expression of different proteins and conversion of LC3B-II were determined by Western blotting. [file Image2.TIF]

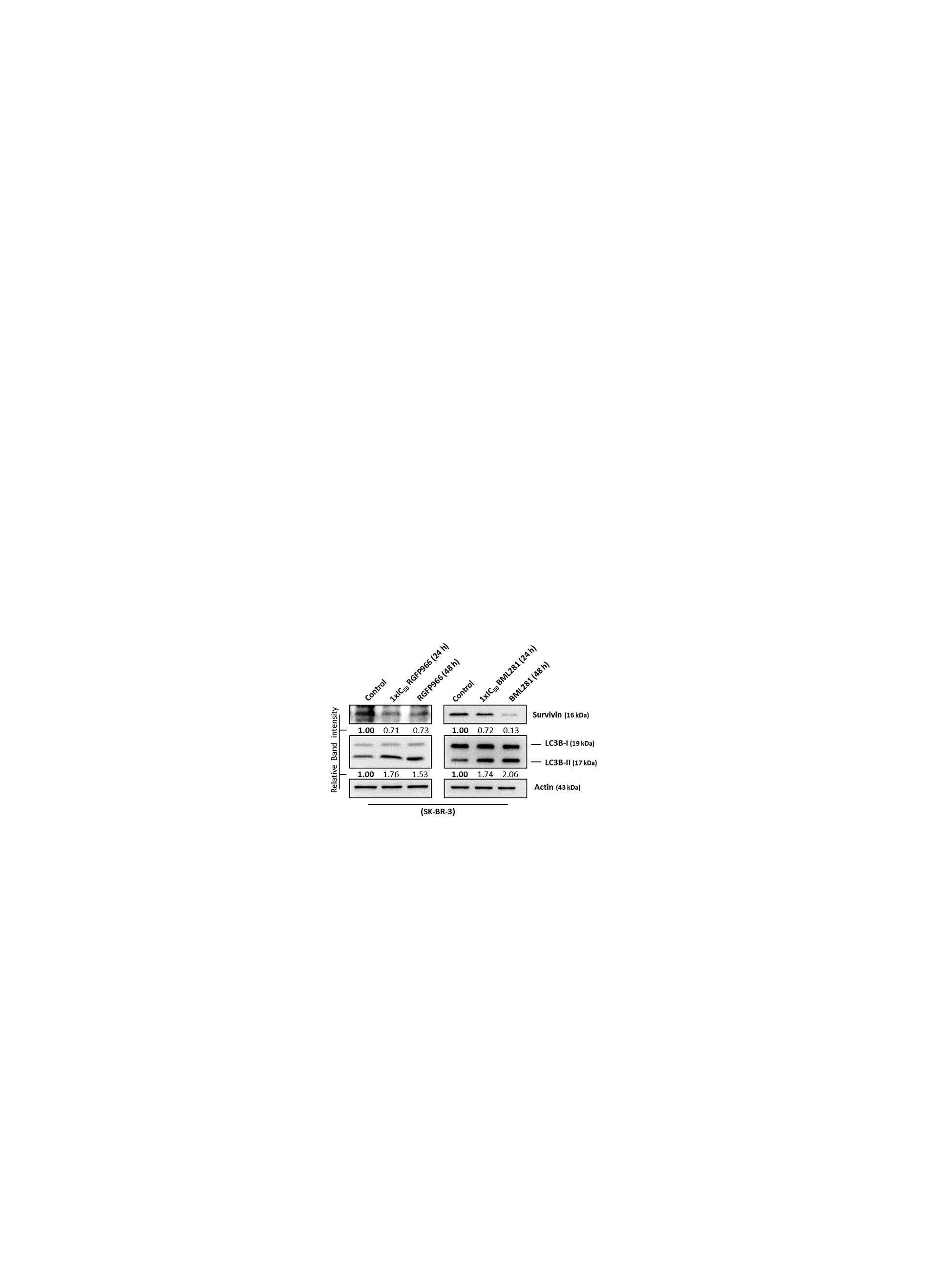

Supplement: Supplementary Figure 3 — Inhibiting HDAC3 and HDAC6 decreases survivin expression and increases LC3B-II conversion in Sk-Br-3 cells. Sk-Br-3 breast cancer cells were treated with either RGFP966 or BML281 and the expression of various proteins was determined by Western blotting. [file Image3.TIF]
